# Supplementary material for: Host-induced spermidine production in motile Pseudomonas aeruginosa triggers phagocytic uptake
Source: eLife. 2020 Sep 22;9:e55744. doi: 10.7554/eLife.55744 (PMC7538158; doi:10.7554/eLife.55744)
Supplement: Figure 1—source data 2. [file elife-55744-fig1-data2.pdf]

Figure 1B – Source File Scanning Electron microscopy

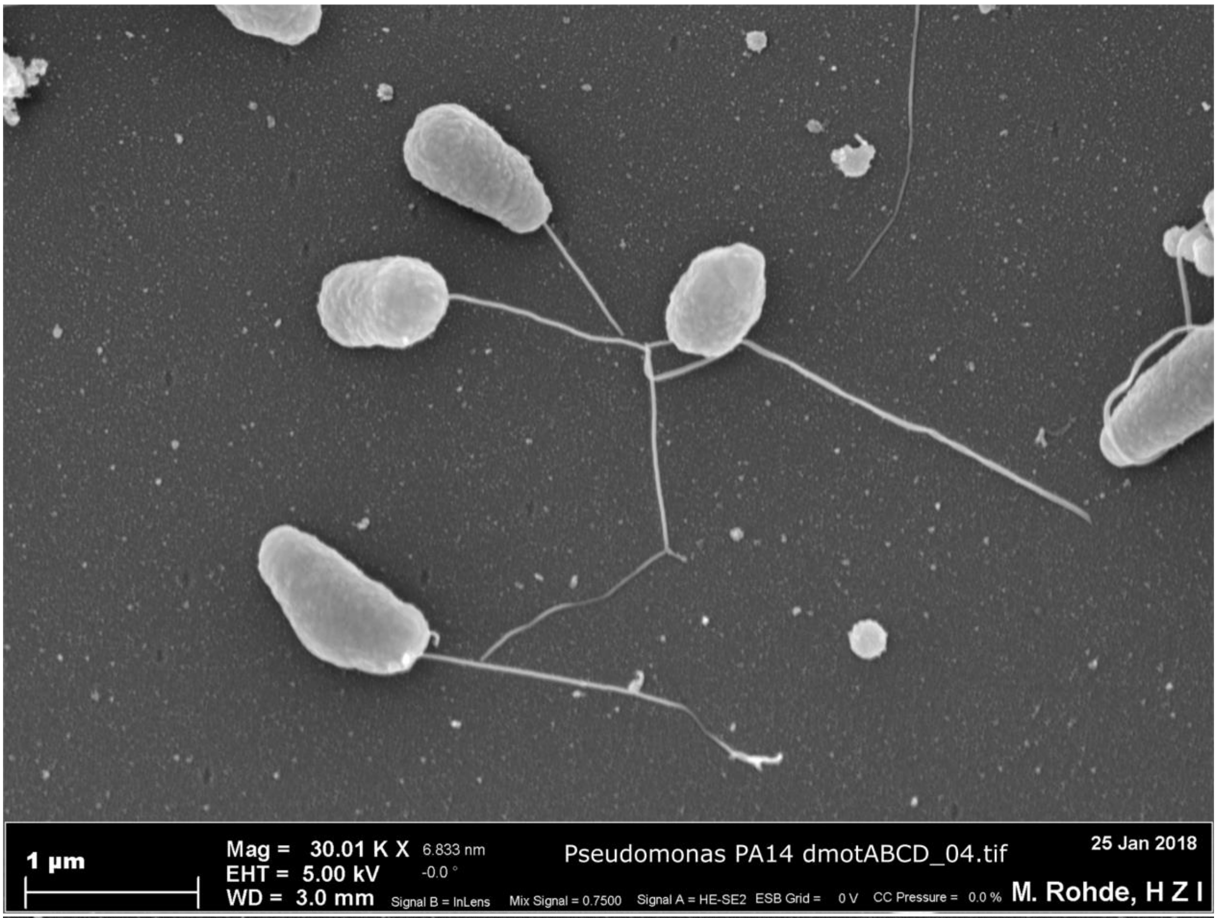

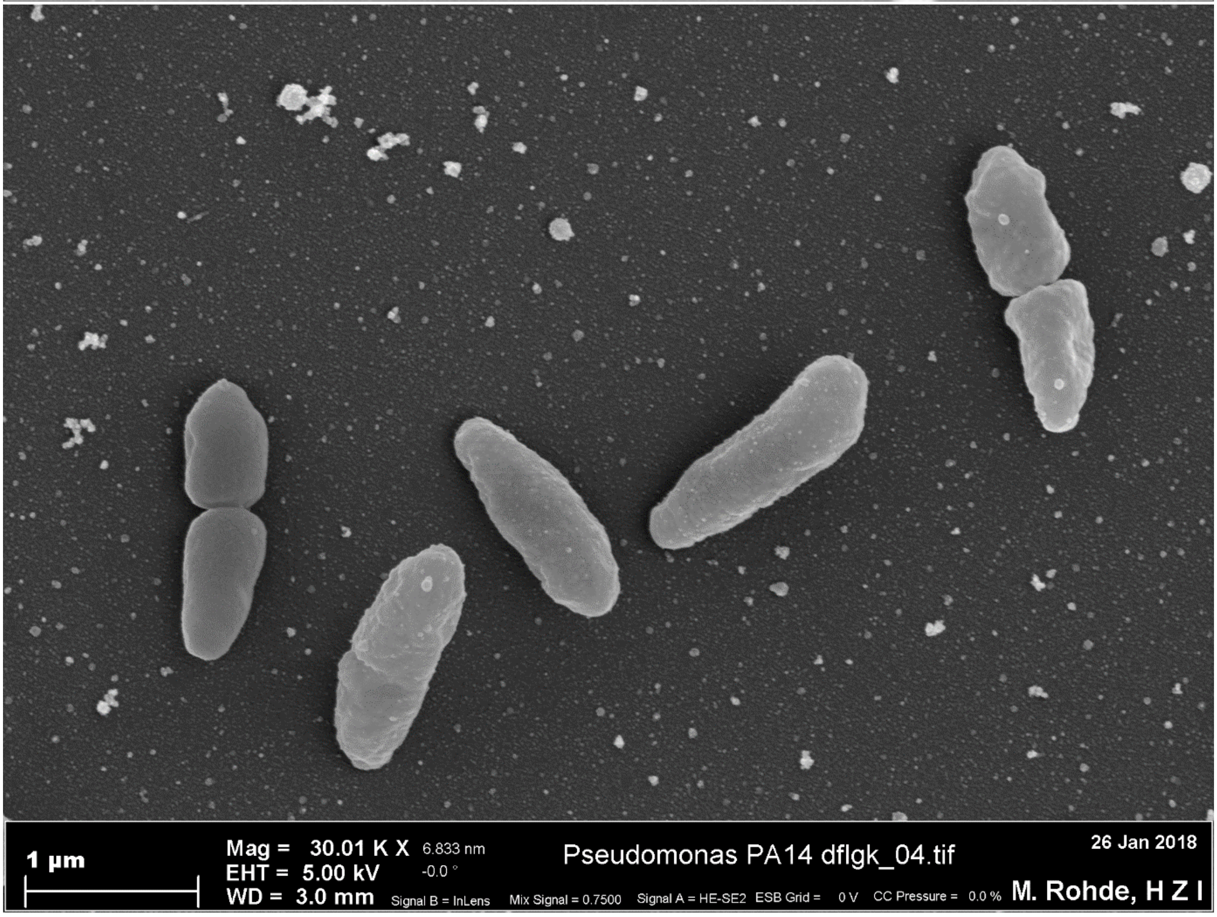

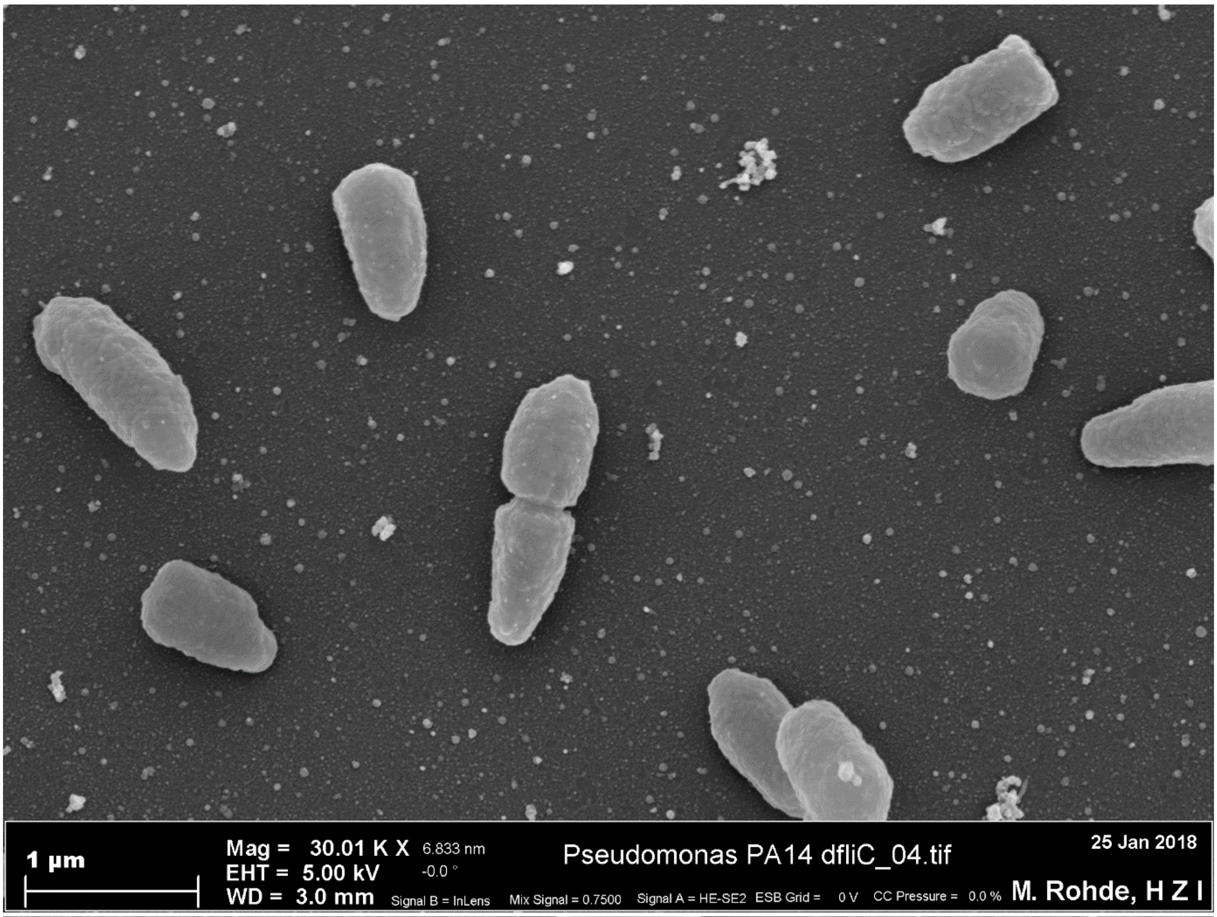
1 µm
Mag = 30.01 K X
6.833 nm
EHT = 5.00 kV
-0.0 °
WD = 3.0 mm
Signal B = InLens
Mix Signal = 0.7500
Signal A = HE-SE2
ESB Grid = 0 V
CC Pressure = 0.0 %
Pseudomonas PA14 dfliC_04.tif
25 Jan 2018
M. Rohde, H Z I

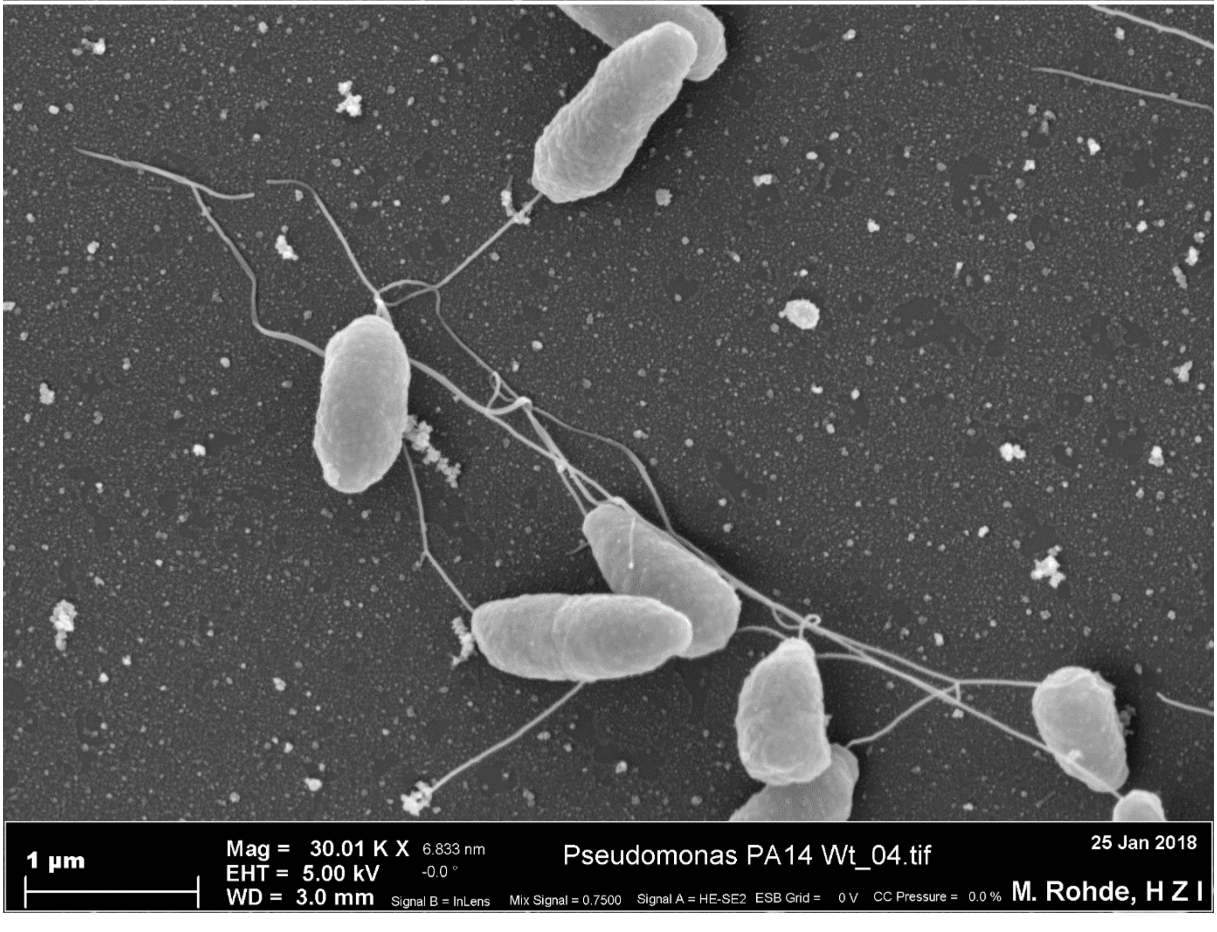
1 µm
Mag = 30.01 K X
6.833 nm
EHT = 5.00 kV
-0.0 °
WD = 3.0 mm
Signal B = InLens
Mix Signal = 0.7500
Signal A = HE-SE2
ESB Grid = 0 V
CC Pressure = 0.0 %
Pseudomonas PA14 Wt_04.tif
25 Jan 2018
M. Rohde, H Z I
