# Supplementary material for: Host-induced spermidine production in motile Pseudomonas aeruginosa triggers phagocytic uptake
Source: eLife. 2020 Sep 22;9:e55744. doi: 10.7554/eLife.55744 (PMC7538158; doi:10.7554/eLife.55744)
Supplement: Figure 1—source data 3. [file elife-55744-fig1-data3.pdf]

Figure 1C_Motility Plates

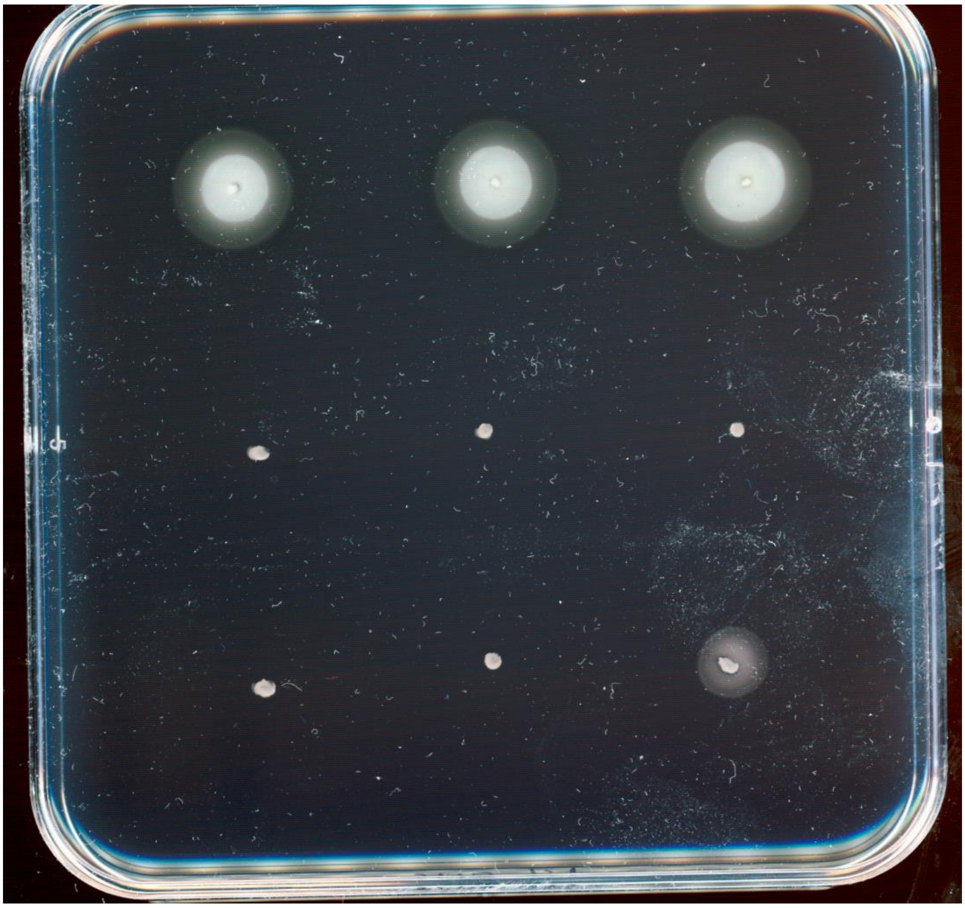

Setup:

Upper lane: PA14 Wt

Middle lane: ***DflgK, DfliC, DPA14_42950***

Bottom lane: ***DmotABCD, DmotABCD, DpilA***
