# Supplementary figures and images for: Host-induced spermidine production in motile Pseudomonas aeruginosa triggers phagocytic uptake
Source: eLife. 2020 Sep 22;9:e55744. doi: 10.7554/eLife.55744 (PMC7538158; doi:10.7554/eLife.55744)

**Figure 2\_ Negative Stain**

**CH2677:**

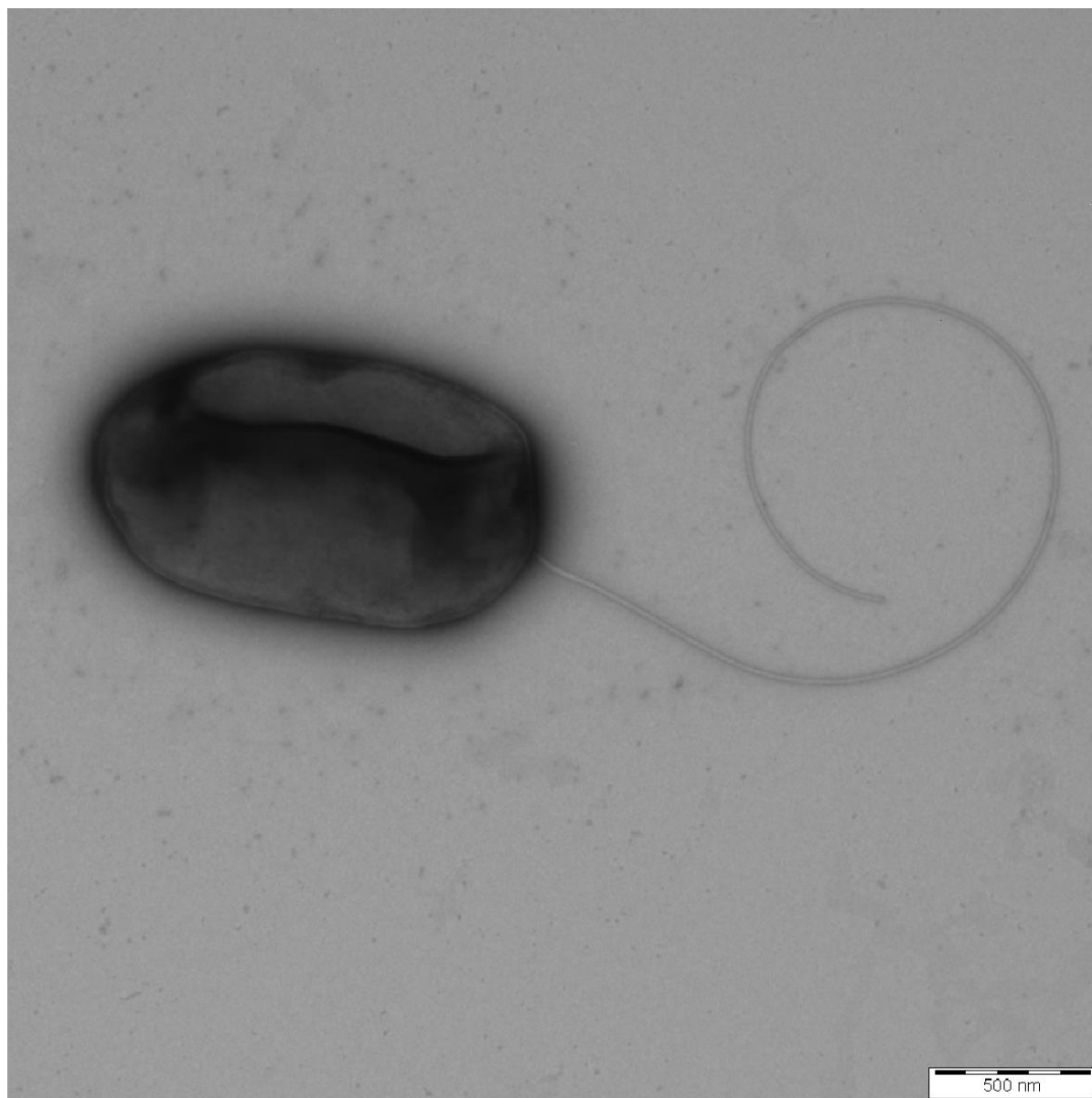

CH3797

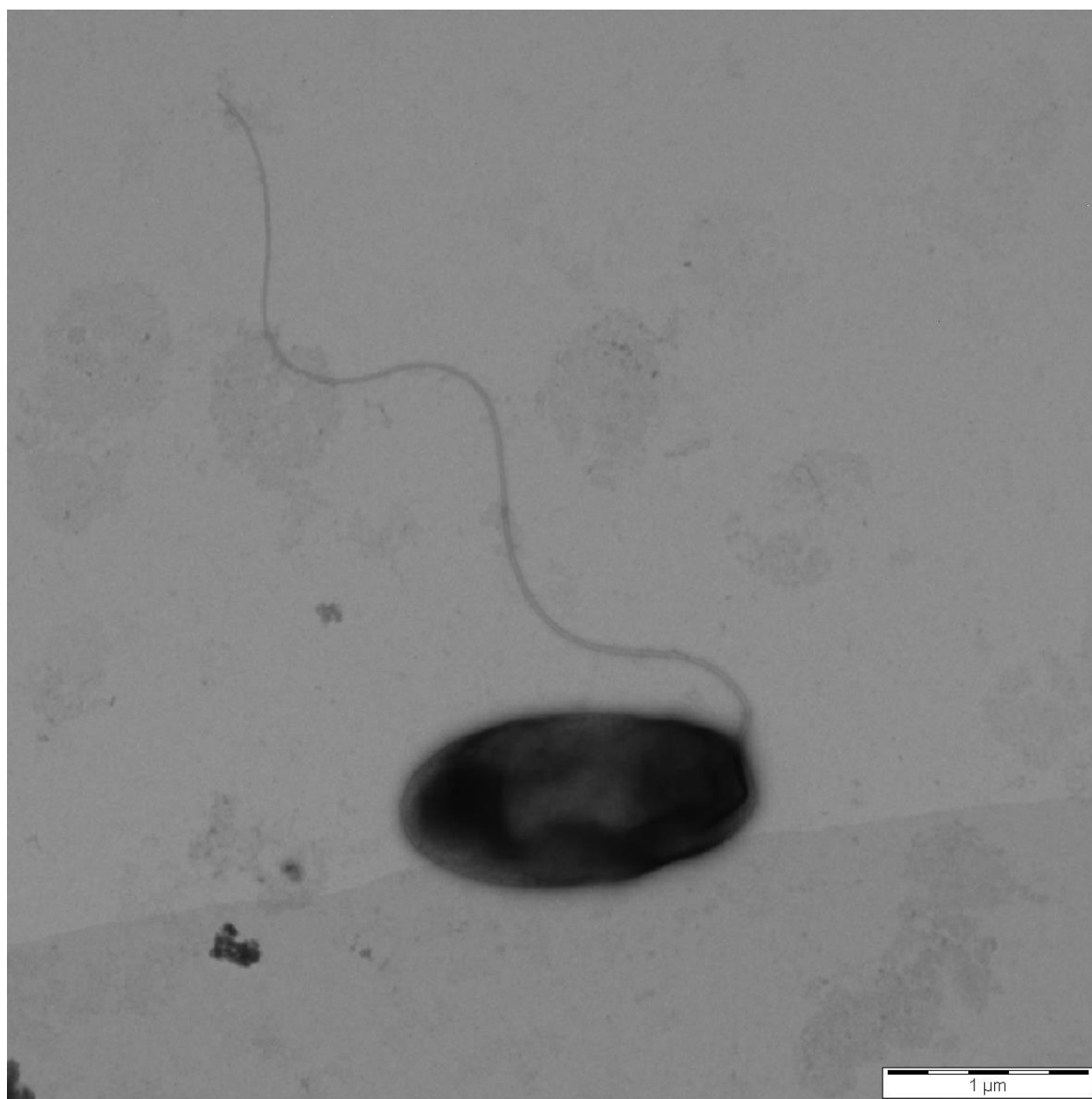

CH5528

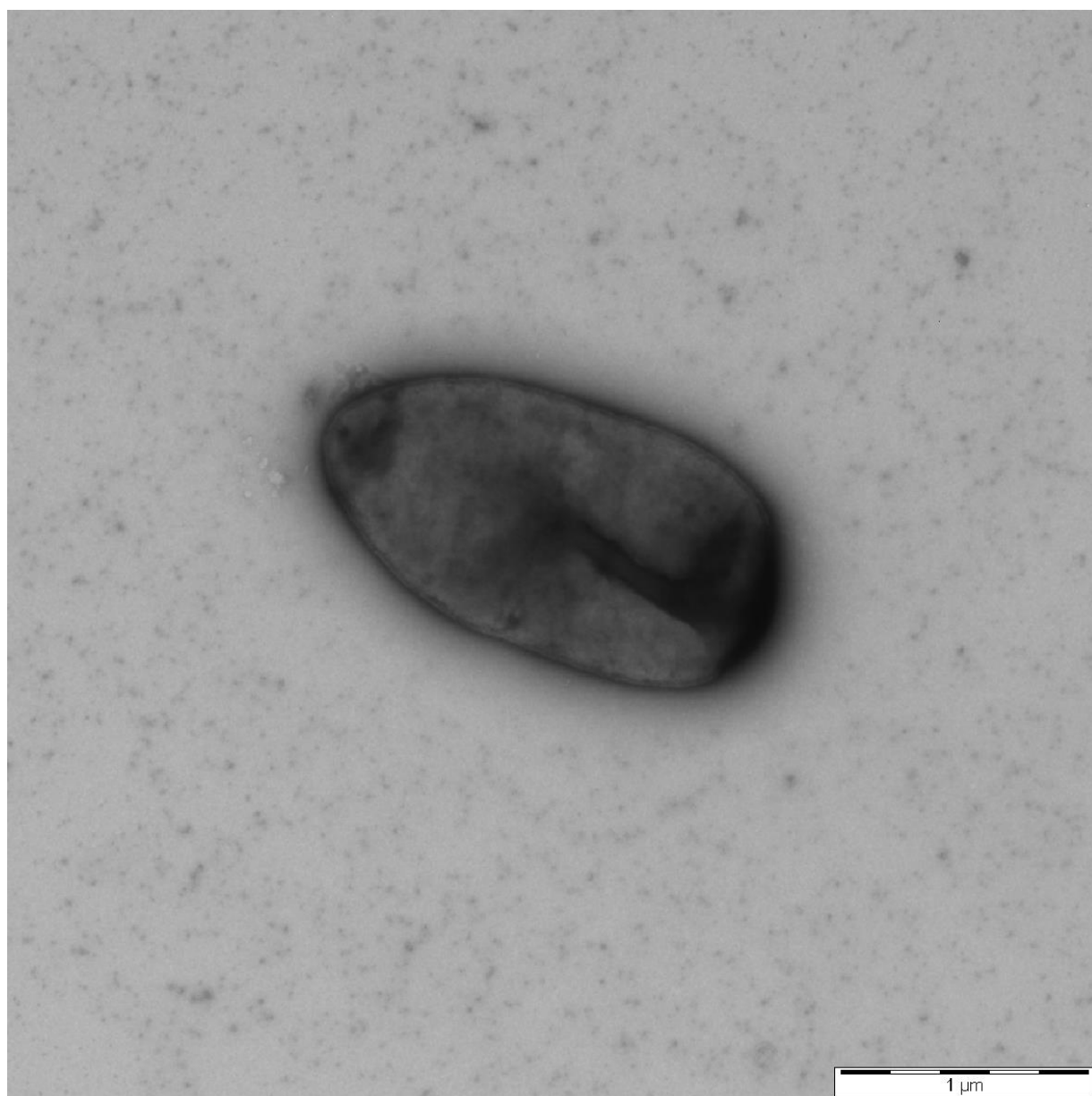

Supplement: Figure 2—source data 1. [file elife-55744-fig2-data1.pdf]
