# Supplementary material for: Host-induced spermidine production in motile Pseudomonas aeruginosa triggers phagocytic uptake
Source: eLife. 2020 Sep 22;9:e55744. doi: 10.7554/eLife.55744 (PMC7538158; doi:10.7554/eLife.55744)
Supplement: Transparent reporting form [file elife-55744-transrepform.pdf]

eLIFE
1st Floor
24 Hills Road
Cambridge CB2 1JP, UK
P 01223 855340
W elifesciences.org
T @elife

eLife's transparent reporting form

We encourage authors to provide detailed information *within their submission* to facilitate the interpretation and replication of experiments. Authors can upload supporting documentation to indicate the use of appropriate reporting guidelines for health-related research (see EQUATOR Network), life science research (see the BioSharing Information Resource), or the ARRIVE guidelines for reporting work involving animal research. Where applicable, authors should refer to any relevant reporting standards documents in this form.

If you have any questions, please consult our Journal Policies and/or contact us: editorial@elifesciences.org.

Sample-size estimation

- You should state whether an appropriate sample size was computed when the study was being designed
- You should state the statistical method of sample size computation and any required assumptions
- If no explicit power analysis was used, you should describe how you decided what sample (replicate) size (number) to use

Please outline where this information can be found within the submission (e.g., sections or figure legends), or explain why this information doesn't apply to your submission:

Sample size was determined based on previous experience and standards for the used experiments.

For cell culture experiments, biological replicates were pooled beforehand to minimize biological variability. For instance, three biological replicates were pooled and treated as one representative sample during the dual-seq analysis

Replicates

- You should report how often each experiment was performed
- You should include a definition of biological versus technical replication
- The data obtained should be provided and sufficient information should be provided to indicate the number of independent biological and/or technical replicates
- If you encountered any outliers, you should describe how these were handled
- Criteria for exclusion/inclusion of data should be clearly stated
- High-throughput sequence data should be uploaded before submission, with a private link for reviewers provided (these are available from both GEO and ArrayExpress)

Please outline where this information can be found within the submission (e.g., sections or figure legends), or explain why this information doesn't apply to your submission:

eLife Sciences Publications, Ltd is a limited liability non-profit non-stock corporation incorporated in the State of Delaware, USA, with company number 5030732, and is registered in the UK with company number FC030576 and branch number BR015634 at the address 1st Floor, 24 Hills Road, Cambridge CB2 1JP | August 2014

All *in vitro* assays (without cells) were performed with biological replicates as denoted in the pictures. Each experiment was repeated at least twice on separate days and the data was pooled as shown in the Figures.

All cell culture experiments were performed in biological triplicates. Each experiment was repeated at least twice on separate days and the representative data is shown in the Figures.

For the dual-seq analysis, three biological replicates were pooled and treated as one representative sample. In total, two pooled samples were sequenced for each strain and analyzed as described in the Material and Method section.

The ELISA measurements was performed on biological triplicates. Each biological triplicate was measured twice as technical replicate.

For biological replication, both, cell cultures and bacteria were independently grown.

All replicas are denoted in the Materials & Methods section as well as in the figure legends. Where applicable, the figures contain individual data points for each replicate.

Transcriptome data are available in the public repository Gene Expression Omnibus (GEO) with the accession number GSE141757:
https://www.ncbi.nlm.nih.gov/geo/query/acc.cgi?acc=GSE141757

Statistical reporting

- Statistical analysis methods should be described and justified
- Raw data should be presented in figures whenever informative to do so (typically when N per group is less than 10)
- For each experiment, you should identify the statistical tests used, exact values of N, definitions of center, methods of multiple test correction, and dispersion and precision measures (e.g., mean, median, SD, SEM, confidence intervals; and, for the major substantive results, a measure of effect size (e.g., Pearson's r, Cohen's d)
- Report exact p-values wherever possible alongside the summary statistics and 95% confidence intervals. These should be reported for all key questions and not only when the p-value is less than 0.05.

Please outline where this information can be found within the submission (e.g., sections or figure legends), or explain why this information doesn't apply to your submission:

Statistical methods, Number of replicates and p-value definitions are described in the according figure legend and the Material and Method section. Furthermore, N is always indicated.

(For large datasets, or papers with a very large number of statistical tests, you may upload a single table file with tests, Ns, etc., with reference to sections in the manuscript.)

Group allocation

- Indicate how samples were allocated into experimental groups (in the case of clinical studies, please specify allocation to treatment method); if randomization was used, please also state if restricted randomization was applied
- Indicate if masking was used during group allocation, data collection and/or data analysis

Please outline where this information can be found within the submission (e.g., sections or figure legends), or explain why this information doesn't apply to your submission:

Additional data files ("source data")

- We encourage you to upload relevant additional data files, such as numerical data that are represented as a graph in a figure, or as a summary table
- Where provided, these should be in the most useful format, and they can be uploaded as "Source data" files linked to a main figure or table
- Include model definition files including the full list of parameters used
- Include code used for data analysis (e.g., R, MatLab)
- Avoid stating that data files are "available upon request"

Please indicate the figures or tables for which source data files have been provided:

eLife Sciences Publications, Ltd is a limited liability non-profit non-stock corporation incorporated in the State of Delaware, USA, with company number 5030732, and is registered in the UK with company number FC030576 and branch number BR015634 at the address 1st Floor, 24 Hills Road, Cambridge CB2 1JP | August 2014

Figure 1 – Source Data: Bacterial strains and plasmids used in this study

Figure 1B – Source Data: Scanning Electron microscopy

Figure 1C – Source Data: Motility Plates

Figure 2D – Source Data: Negative Stain

Figure 3 - Source Data: Summary of the 20 differentially expressed genes that are shared by the motility mutants in comparison to PA14 Wt.

Figure 3B - Source Data: Significantly enriched host pathways

Figure 3 - Figure Supplement 4 - Source Data: Enriched functions in the *Pseudomonas* variants

Figure 4 - Source Data 1: Bacterial gene regulation

Figure 4 - Source Data 2: Positive interspecies correlation of 53 PA14 genes and their association with 74 host PIP3 genes

Figure 4 - Source Data 3: Negative interspecies correlation of 53 PA14 genes and their association with 74 host PIP3 genes

Figure 5 - Source Data: Enrichment of calcium and PIP3-related functions of a downregulated (log2FC < 0) subset of Top 1000 genes from ΔfliC, ΔflgK and ΔmotABCD mutants

Figure 6 - Source Data 1: Spermidine induced gene induction

Figure 6 - Source Data 2: Shared enriched functions of spermidine-treated and PA14-infected macrophages
